# A Novel PD-L1-Containing MSLN Targeting Vaccine for Lung Cancer Immunotherapy

**DOI:** 10.3389/fimmu.2022.925217

**Published:** 2022-06-20

**Authors:** Wuyi Zeng, Jiayi Pan, Zixuan Fang, Jiangtao Jia, Rong Zhang, Menghua He, Hanyu Zhong, Jiashan He, Xinyu Yang, Yi Shi, Bei Zhong, Jun Zeng, Bishi Fu, Maoping Huang, Hui Liu

**Affiliations:** ^1^School of Basic Medical Sciences, The Sixth Affiliated Hospital of Guangzhou Medical University, Qingyuan People’s Hospital, Guangzhou Medical University, Guangzhou, China; ^2^The State Key Laboratory of Respiratory Disease, Guangdong Provincial Key Laboratory of Allergy and Clinical Immunology, Guangzhou, China

**Keywords:** dendritic cells, MSLN, PD-L1, immunotherapy, therapeutic vaccine

## Abstract

Therapeutic tumor vaccines have become an important breakthrough in the treatment of various solid tumors including lung cancer. Dendritic cells (DCs)-based tumor vaccines targeting tumor-associated antigens (TAAs) play a key role in immunotherapy and immunoprevention. However, the weak immunogenicity of TAAs and low immune response rates are a major challenge faced in the application of therapeutic tumor vaccines. Here, we tested whether targeting an attractive target Mesothelin (MSLN) and PD-L1 immune checkpoint molecule to DCs *in vivo* would elicit therapeutic antitumor cytotoxic T lymphocyte (CTL) response. We generated specific MSLN fragment combined with PD-L1 and GM-CSF peptide immunogen (MSLN-PDL1-GMCSF) based on the novel anti-PD-L1 vaccination strategy we recently developed for the cancer treatment and prevention. We found that DCs loaded with MSLN-PDL1-GMCSF vaccine elicited much stronger endogenous anti-PD-L1 antibody and T cell responses in immunized mice and that antigen specific CTLs had cytolytic activities against tumor cells expressing both MSLN and PD-L1. We demonstrated that vaccination with MSLN-PDL1-GMCSF potently inhibited the tumor growth of MSLN^+^ and PD-L1^+^ lung cancer cells, exhibiting a significant therapeutic anti-tumor potential. Furthermore, PD-1 blockade further improved the synergistic antitumor therapeutic efficacy of MSLN-PDL1-GMCSF vaccine in immunized mice. In summary, our data demonstrated for the first time that this PD-L1-containing MSLN therapeutic vaccine can induce persistent anti-PD-L1 antibody and CTL responses, providing an effective immunotherapeutic strategy for lung cancer immunotherapy by combining MSLN-PDL1-GMCSF vaccine and PD-1 blockade.

## Introduction

Lung cancer is the most common malignant tumor with high morbidity and mortality in the world. Surgical treatment alone is no longer effective in further improving survival rates, and effective treatment of lung cancer remains a major challenge to be faced (1–3). Dendritic cells (DCs) are specialized antigen-presenting cells (APCs) that initiate and modulate innate and adaptive immunity (4, 5). DCs can present antigen through MHC I and MHC II molecules and activate CD4+T and CD8+T, which can activate specific anti-tumor immune response, so as to make tumor recede. Therefore, DC can be used to prepare therapeutic anti-tumor vaccine (6–8). Sipuleucel-T (Provenge), the first DCs-based cancer vaccine, which was approved by the US Food and Drug Administration (FDA) in 2010, was exploited for resistant prostate cancer (9). For the past two decades, DC therapy has been indicated to be able to induce anti-tumor immunity, which is safe and well-tolerated (10, 11). Programmed death 1 (PD-1) and programmed death ligand 1 (PD-L1) are the most well-studied immune checkpoints in recent years. Many tumor cells, including lung, ovarian, melanoma and pancreatic tumors, evade immune surveillance by upregulating PD-L1 expression (12, 13). The binding of PD-1 to PD-L1 leads to suppression of tumor-specific T cell immune responses (14). Currently, PD-1/PD-L1 monoclonal antibodies have been developed as immune checkpoint inhibitors for cancer therapy to remove the “brake” on the immune system and restore the ability of T cells to attack tumor cells (15). Nevertheless, treatment with anti-PD-1/PD-L1 antibodies has produced long-lasting and effective antitumor response in only a small percentage of patients (16–18). DCs or antigen-loaded DCs can directly induce antibody responses and promote antibody production of CD40-activated naive and memory B cells during stimulation of B cell responses (19). Moreover, our recent studies also found that DCs can activate both cellular and humoral immune responses (13). Therefore, identifying predictive biomarkers and designing rational PD-(L)1-based combination therapies has become the focus of cancer immunotherapy.

Mesothelin (MSLN) is a glycosylphosphatidylinositol-linked membrane glycoprotein which is highly expressed in a variety of tumors and is also expressed in mesothelial cells of healthy individuals, but at low levels. Therefore, it can be considered as a promising target protein for tumor-targeted therapy (20–22). In the tumor environment, MSLN plays an important role in survival, proliferation, and migration/invasion of cancer cells as well as in drug resistance (23). Over the years, evidence has accumulated with regards to the importance of MSLN as a tumor-associated antigen (TAA) overexpressed in almost one-third of human cancers. For these reasons, various types of anti-MSLN therapies have been developed, including antibodies, antibody-drug conjugates (ADCs), immunotoxins, cancer vaccines, and chimeric antigen receptor (CAR)-T cell immunotherapies (24–26). Granulocyte macrophage colony stimulating factor (GM-CSF), produced mainly by macrophages and activated T cells, is a cytokine with multiple biological activities (27). GM-CSF is a key cytokine essential for the differentiation, proliferation, and recruitment of DCs and promotes their capacity for antigen presentation, co-stimulatory molecule expression, and proinflammatory cytokine production. Therefore, it has been used as an adjuvant in several cancer vaccines to boost DC-mediated antitumor immunity (28–30). Preclinical studies have shown that GM-CSF not only has the capacity to increase antigen-induced immune responses, but it also can alter the Th1/Th2 cytokine balance. It appears that GM-CSF can stimulate both Th1 and Th2 type responses depending on immune cells and cytokines in the immediate local environment (31, 32). Therefore, GM-CSF acts an essential role as immune adjuvant played in tumor vaccine for enhancing effective immune response.

In this study, we generated a new therapeutic vaccine (MSLN-PDL1-GMCSF), with both autonomous induction of anti-PD-L1 antibody production and MSLN-specific targeting characteristics. We found that this MSLN-PDL1-GMCSF protein-loaded DCs vaccine induced an effective antigen-specific anti-tumor CTL response *in vivo*, and showed significant therapeutic effects in tumor-bearing mice. Furthermore, combination therapy with PD-1 blockade produced synergistic antitumor effects, which provides a new effective strategy for immunotherapy of solid tumors.

## Materials and Methods

### Mice and Cell Lines

Six- to eight-week-old male C57BL/6 mice were purchased from Charles River Laboratories and bred at Guangzhou Medical University under specific pathogen-free (SPF) conditions. All animal procedures were approved by the Animal Ethics Committee of Guangzhou Medical University. The mice Lewis lung carcinoma (LLC) cell line was purchased from ATCC (American Type Culture Cell Bank). Cell lines were maintained in DMEM (Gibco) supplemented with 10% FBS (ExCell), 1% Penicillin-Streptomycin (Gibco). LLC stably expressing human MSLN and PD-L1 were maintained in DMEM supplemented with 2.5μg/ml puromycin (Solarbio).

### Protein Production and Purification

To obtain the PDL1-GMCSF and MSLN-PDL1-GMCSF vaccines, the fusion gene of human MSLN extracellular domain, PD-L1 extracellular domain, T helper epitope and GM-CSF sequence was synthesized and cloned into pET21a expression vector to construct pET-21a-MSLN-PDL1-GMCSF and pET-21a-PDL1-GMCSF expression plasmids, respectively. Then the plasmids were transformed into BL21(DE3) expressing strain and induced to express in inclusion body form under IPTG (0.1 mM) condition. Next, the proteins were purified using Ni-NTA columns according to the instructions of the kit (Abbkine#KTP20010). The purified proteins solution was then subjected to gradient dialysis and endotoxin removal, and the entire proteins production process were analyzed by SDS-PAGE and Western blot. The prepared proteins were stored at -80°C for further studies. In addition, recombinant human PD-L1 protein was purchased from Abcam company.

### Lentiviral-Transduced Tumor Cell Lines

Lentiviral vectors expressing MSLN and PD-L1 and carrying puromycin resistance genes were constructed using a three-plasmid system (psPAX2, PMD2.G, pHBLV™) for lentiviral packaging. Mass de-endotoxin extraction of plasmids was performed using Qiagen extraction kit. 293T cells were transfected according to the instructions of LipoFiter™ reagent, and virus supernatants were collected twice at 48h and 72h after transfection. After virus resuspension, LLC cells were infected, and polybrene with a final concentration of 5μg/mL was added to improve infection rate. After 24 hours, the solution was changed, and the optimal MOI was 30. After 48 hours, fresh complete culture medium containing puromycin (5μg/mL) was added to screen stable LLC cell lines. The cells were collected for further Real-time Quantitative PCR (qPCR), Western blot and flow cytometry analysis.

### Preparation of Dendritic Cells

C57BL/6 mouse bone marrow (BM)-derived dendritic cells (DCs) were prepared as we described previously (13). In brief, mouse BM was flushed from leg bones, and depleted of red cells with Red Cell Lysis Solution (Biosharp). Cells were cultured in RPMI-1640 supplemented with 10% FBS, recombinant mouse GM-CSF/ml (20ng/ml) and recombinant mouse IL-4 (10ng/ml; PeproTech). Every other day, the supernatant was replaced at half volume with fresh media containing adequate cytokines. On day 7, recombinant proteins (PDL1, PDL1-GMCSF, MSLN-PDL1-GMCSF;100µg/mL) or PBS was added, and then bacterial lipopolysaccharide (LPS; Sigma) was added at 1μg/mL after 4h of antigen loading for DC maturation. After 24h of culture, the expression of characteristic DC-specific markers (CD11c and CD80) as determined by FACS.

### DC Immunization and Tumor Model

C57BL/6 mice were randomly divided into 5 groups (8 mice per group) as follows: 1) PBS control, 2) PDL1, 3) PDL1-GMCSF, 4) MSLN-PDL1-GMCSF, 5) MSLN-PDL1-GMCSF + anti-PD-1. 2x10^5^ LLC-MSLN-PDL1 cells at exponential growth stage were subcutaneously injected into the right side of the mouse. After 7 days, the mice were immunized with 1x10^6^ antigen-loaded dendritic cells *via* footpad injection, twice at one-week intervals. Tumor size was measured with a vernier caliper every three or five days. The tumor volume was calculated as follows: (longest diameter) × (shortest diameter)^2^×0.5. Images were taken every 7 days with a small animal live imager (IVIS Lumina XRMS Series III). The method of living image was as follows: mice were anesthetized generally and 150mg D-Luciferin was intraperitoneally injected into mice per kg, and then imaging analysis was performed after 10-15 minutes of injection.

### Intracellular Staining (ICS) and Flow Cytometry Analysis

Spleens were isolated from immunized C57BL/6 mice 3 days after the last DC administration (n=3). Prepared splenocytes (1×10^6^ cells/well) were restimulated in 24-well plates with 5 µg/mL freshly-prepared vaccine protein for 6 h in the presence of recombinant mouse IL-2 (20ng/ml; PeproTech). 50ng/mL PMA, 1ug/ml Ionomycin and 10 µg/mL Brefeldin A (Absin) was added to accumulate intracellular cytokines. After restimulation, the cells were firstly incubated with anti-mouse CD3, CD4 and CD8 antibodies for surface staining. Subsequently, intracellular staining for IL-2, IFN-γ, Granzyme B and Perforin were performed after these cells were fixed and permeabilized. Fixable viability dye was used to gate out dead cells. Data were collected on FACS verse (BD Biosciences) and analyzed with Flow Jo software. The antibodies used in this study were including FITC anti-mouse CD3 (Thermo Fisher), PE anti-mouse CD4 (BD Biosciences), PerCP-Cy™5.5 anti-mouse CD8a (BD Biosciences), PE-Cy7 anti-mouse IL-2 (BD Biosciences); BV510 anti-mouse IFN-γ (Biolegend); BV421 anti-mouse Granzyme B (Biolegend); APC anti-mouse Perforin (Biolegend); Fixable Viability Stain 780 (BD Biosciences); Fixation/Permeabilization Kit (BD Biosciences).

### Antibody ELISA Assay

After immunization, serum of 3 mice in each group was collected for ELISA detection. ELISA plates were coated with recombinant PD-L1 proteins (500ng/mL) overnight at 4°C. The next day, the PD-L1-coated plates were blocked with BSA, and then added with serial dilutions of serum, and incubated at room temperature for 2h. After extensive washes, HRP-labeled anti-mouse IgG antibody (Bioss) was added and incubated at room temperature for 1 h. Then TMB was added to detect ELISA reactions. Optical density (OD) was read at 450 nm on a multi-plate reader (Varioskan Flash, Thermo).

### Hematoxylin-Eosin (HE) Staining

Mice livers and kidneys (n=3) were isolated from the above immune groups and fixed in 4% paraformaldehyde. After dewaxing treatment, stain sections with Hematoxylin solution for 3-5 min, rinse with tap water. Then treat the section with Hematoxylin Differentiation solution, rinse with tap water. Treat the section with Hematoxylin Scott Tap Bluing, rinse with tap water. 85% ethanol for 5 min; 95% ethanol for 5 min; then stain sections with Eosin dye for 5 min. Finally sealed with neutral gum for dehydration. Observe with microscope inspection, image acquisition and analysis.

### Statistical Analysis

All analysis was performed using GraphPad Prism 8.0 statistical software. Two-way ANOVA and log-rank (Mantel-Cox) tests were used to analyze the tumor growth and mice survival data, respectively. All the other data were analyzed using unpaired two-tailed t tests. A value of p < 0.05 was considered statistically significant.

## Results

### Production of Recombinant Protein Immunogens

**Figure 1:**
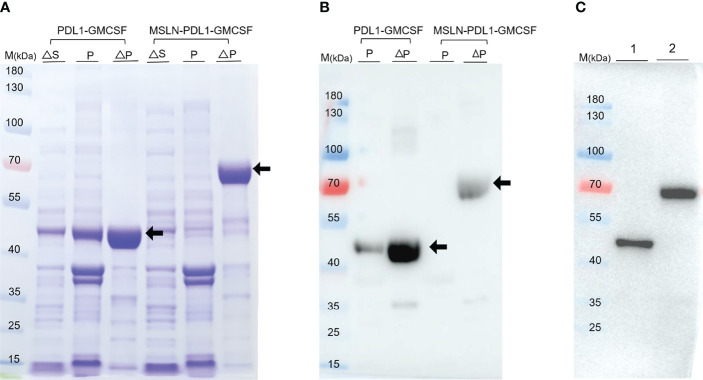
Expression and purification of the recombinant protein PDL1-GMCSF and MSLN-PDL1-GMCSF. **(A)** The fusion protein consisting of human MSLN, PD-L1, Th epitope sequence and GM-CSF was designed. The recombinant protein PDL1-GMCSF and MSLN-PDL1-GMCSF in pellet and supernatant were inducted upon IPTG (0.1 mM) or IPTG-free at the expected size using SDS-PAGE analysis. **(B)** Western blot analysis of the purified protein PDL1-GMCSF and MSLN-PDL1-GMCSF using His-tagged antibody was shown. **(C)** Western blot analysis of the dialyzed proteins PDL1-GMCSF (lane 1) and MSLN-PDL1-GMCSF (lane 2) was performed with His-tagged antibody. M, Marker; S, Supernatant; P, Pellet; ∆, Induction by IPTG.

A key problem in tumor immunotherapy is the weak immunogenicity and low immune response rate of tumor antigen (10). To generate new immunogens for cancer treatment and prevention, two specific PD-L1-containg peptides fusion proteins PDL1-GMCSF and MSLN-PDL1-GMCSF were designed and synthesized based on the recent method of peptide assembly we developed (13) and cloned them into the pET21a expression vector. Plasmid construction map is shown in **Supplementary Figure 1**. Then, the plasmids were transformed into BL21 (DE3) (*E. coli*) to express the recombinant proteins PDL1-GMCSF and MSLN-PDL1-GMCSF, respectively. After culture fermentation and induction with IPTG, cells were collected, lysed, and examined by SDS-PAGE. As indicated by the black arrows (**Figure 1A**), the target proteins were expressed in pellet with expected molecular weights of 41.94 kDa (PDL1-GMCSF) and 65.41 kDa (MSLN-PDL1-GMCSF) upon induction. The His-tagged protein vaccine was then purified by Ni-NTA column under denaturing conditions, and the eluted fractions was further analyzed by Western blot using His antibody, which verified the accuracy of the target proteins (**Figure 1B**, arrows). To create a physiological consistency, the elution enriched portion of the purified protein was further dialysis and then analyzed by Western blot (**Figure 1C**). Additional quality control tests for endotoxin, mycoplasma and microorganisms were also performed. The purity of the recombinant proteins PDL1-GMCSF and MSLN-PDL1-GMCSF was >90% and the endotoxin was at an acceptable low level used for further studies.

### Maturation of DCs After Loading With Fusion Protein Vaccines

**Figure 2:**
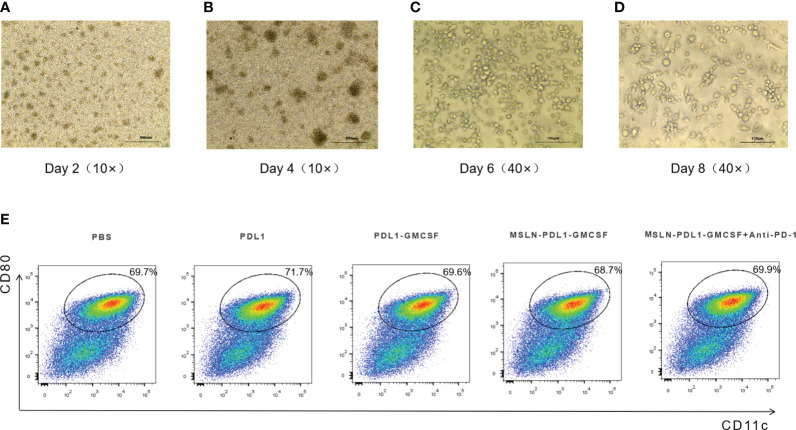
Morphology of murine BMDCs at different days of culture and costimulatory molecule (CD80 and CD11c) expression of murine mature-BMDCs. Murine bone marrow cells were cultured in RPMI-1640 with 10% FBS containing 20ng/ml recombinant mouse GM-CSF and 10ng/ml IL-4 for 8 days. Fresh medium was supplemented every two days. **(A)** Murine bone marrow cells at day 2. **(B)** BMDCs at day-4. **(C)** Immature un-treated-BMDCs at day 6. **(D)** BMDCs were loaded with different fusion proteins (PBS, PDL1, PDL1-GMCSF, MSLN-PDL1-GMCSF) at day 7 and then after 4 hours, stimulated by LPS (1μg/ml) for another 24 hours. **(E)** The expression levels of the maturation-stimulating molecules CD80 and CD11c in BMDCs were analyzed by surface staining and flow cytometry.

Mouse bone marrow-derived DCs were prepared as previously described (13). Approximately 2~4×10^7^ bone marrow cells were obtained per mouse, cultured in an incubator containing 5% CO_2_ at 37°C, denoted as Day 0. At this time, the cells were small in size, mostly round and without obvious protuberance. After 2 days culture *in vitro*, the cell volume increased, some cells were semi-adherent, and a few cell colonies were formed (**Figure 2A**). On day 4, a large number of cells grew in clusters (**Figure 2B**). After 6 days of culture, the immature BMDCs were obtained with the culture in the medium containing GM-CSF. Clusters of colonies of BMDCs were formed, and amounts of floating and semi-adherent BMDCs were seen. The cell volume was larger than before, with round or shuttle shape, and some cells were visible as spines (**Figure 2C**). After 24h stimulation with LPS (1μg/mL), the number of cells increased, and a large number of DCs with typical morphology were released from the colonies with obvious dendritic protrusions (**Figure 2D**). In addition, in order to detect the maturity of BMDCs, on day 7 of culture, DCs were individually loaded with PDL1, PDL1-GMCSF, MSLN-PDL1-GMCSF protein and PBS. After 4h of antigen loading, LPS was added and stimulated overnight. Surface staining and subsequent flow cytometry analysis showed that the majority of DC cells used for immunization were induced to mature, with expression levels of co-stimulatory molecules CD11c and CD80 ranging from 68.7% to 71.7% (**Figure 2E**). The results show that the antigen uptake and DC maturation events have occurred during this culture and stimulation process.

### PD-L1-Containing MSLN Vaccine Induces Effective Th1 Cytokine Secretion and Elevated Anti-PD-L1 Antibody Production

**Figure 3:**
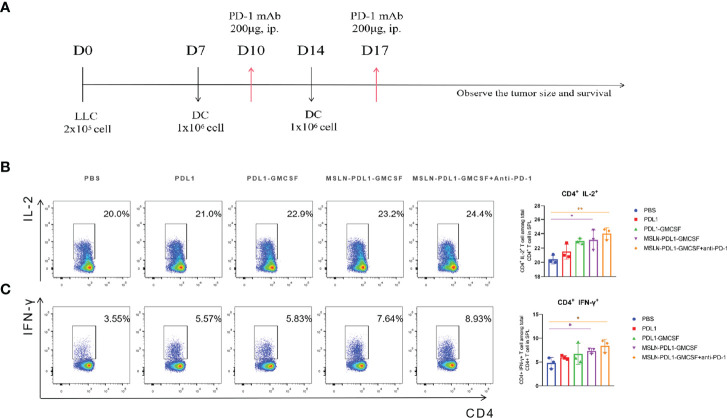
MSLN-PDL1-GMCSF protein vaccine pulsed with DCs increases IFN-γ and IL-2 secretion by Th cells. **(A)** C57BL/6 mice were immunized twice, weekly, with 100μg/mL of vaccine protein-loaded DCs and PBS-DC control. Splenocytes were isolated for assay 3 days after immunization. **(B)** The frequency of IL-2^+^ production in CD4^+^ T cells by intracellular staining analysis and flow cytometry. **(C)** Frequency of IFN-γ^+^ among CD4^+^ T cells was analyzed. Mean ± SD (n=3). *p < 0.05, **p < 0.01.

**Figure 4:**
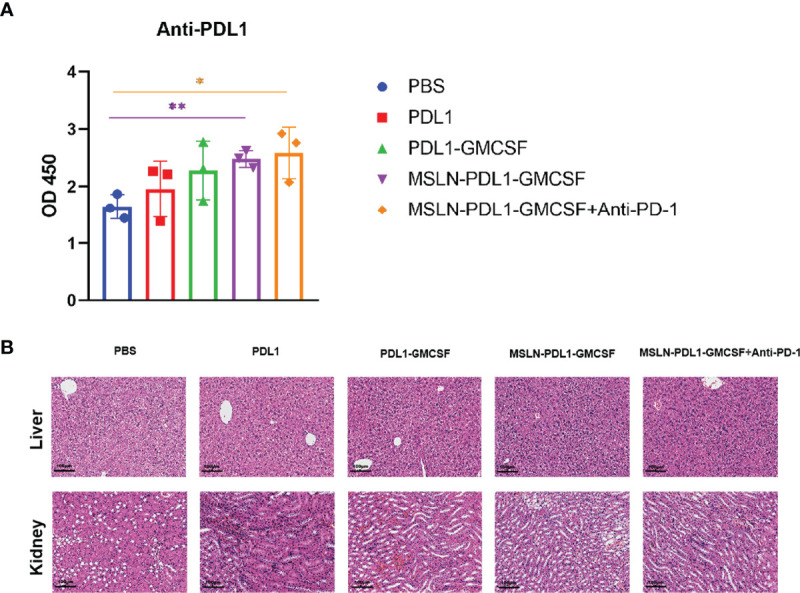
Groups of C57BL/6 mice were immunized with protein-loaded DCs (1×10^6^ cells/mouse) at twice weekly interval, and sera from each group mice were collected 3 days after the end of immunization. **(A)** PD-L1-specific IgG levels from the sera of each group were determined by ELISA. **(B)** HE staining analysis of liver and kidney sections from each group of mice as described in “*Materials and Methods*”. The original magnification of the images is ×10. Mean ± SD (n=3). *p<0.05, **p < 0.01.

To study whether this PD-L1-containing MSLN vaccine can induce efficient immune response, C57BL/6 mice were firstly immunized twice with 100 µg/mL protein-loaded DCs and PBS-DCs weekly. On the third day after immunization with MSLN-PDL1-GMCSF protein-loaded DC vaccine, C57BL/6 mice were intraperitoneally injected with PD-1 monoclonal antibody (McAb) (200μg/mouse) to carry out combination therapy (**Figure 3A**). Three days after the second immunization, spleens of immunized mice were isolated and digested into single-cell suspensions, which were then stimulated in 24-well plates with 50ng/mL PMA, 1ug/ml Ionomycin and 10 µg/mL Brefeldin A for 6 h. CD4^+^ T cells producing IL-2 and IFN-γ were then determined and analyzed by intracellular staining and flow cytometry. As expected, the Th cells immunized with MSLN-PDL1-GMCSF protein vaccine generated a significantly higher percentage of IL-2 compared with cells immunized with PBS-DCs (**Figure 3B**). Similarly, the frequency of IFN-γ producing T cells was induced by a 2.1-fold increase in total CD4^+^ cells, similar to IL-2 induction (**Figure 3C**). These results clearly demonstrated that MSLN-PDL1-GMCSF protein vaccine elicit enhanced IFN-γ and IL-2 production and might generate good capability for CTL induction. Furthermore, to further study whether DCs-loaded with MSLN-PDL1-GMCSF can induce PD-L1-specific antibody responses, we used sera of immunized mice for anti-PD-L1 ELISA detection. As shown in **Figure 4A**, it showed that levels of anti-PD-L1 antibodies (IgG) in sera of MSLN-PDL1-GMCSF loaded DCs immunized mice slightly increased (p<0.05) compared to other two immunized groups, similar to our previous observation immunized with PDL1-Vax (13). Interestingly, when combined with PD-1 McAb treatment, the secretion of cytokines and anti-PD-L1 production were significantly increased determined by FACS and ELISA assay in this test. To further verify the possible adverse autoimmune pathology induced by immunization with protein-loaded DCs, HE staining was performed on sections of vital organs and tissues of immunized mice, and no significant pathological toxicity was observed in immunized mice (**Figure 4B**). Taken together, our data implies that this PD-L1-containing MSLN vaccine can induce effective Th1 responses and anti-PD-L1 antibody production, and it a relatively safe and effective tumor vaccine.

### Vaccination Generates Significantly Enhanced Cytotoxic T Cell Response

**Figure 5:**
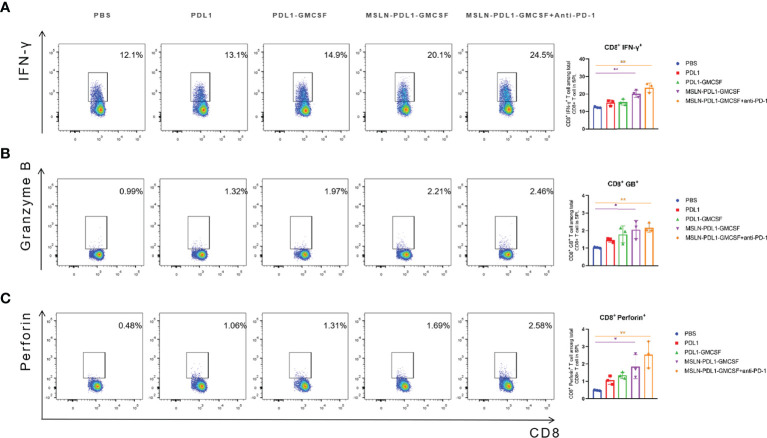
DC vaccination produced a significantly enhanced cytotoxic T cell response. Spleen cells were isolated from each group 3 days after the second inoculation of C57BL/6 mice, and incubated with RPMI-1640 containing 10%FBS for 6 hours, adding PMA (50ng/mL), Brefeldin A(10ug/ml) and Ionomycin(1ug/ml). The cells were surface stained and intracellularly stained with FACS to analyze the expression of IFN-γ **(A)**, granzyme B **(B)** and perforin **(C)** in CD8^+^ T cells. Mean ± SD (n=3). *p < 0.05, **p < 0.01.

In human anti-tumor immune response, cellular immunity is the predominant form, and the production of CD8^+^ cytotoxic T lymphocyte cells (CTL) is the core of effective anti-tumor cell immunity (33). Therefore, to study whether this MSLN-PDL1-GMCSF-loaded DC vaccine can induce an effective antigen-specific CTL response, C57BL/6 mice were immunized twice with fusion protein-loaded DCs and then spleen T cells were obtained 3 days after the second vaccination as described previously. The effector molecules IFN-γ, Granzyme B, and perforin were then tested to evaluate the cytotoxic T cell response. As shown in **Figure 5A**, the increased expression level of IFN-γ was observed in CD8^+^ T cells using intracellular staining and FACS. MSLN-PDL1-GMCSF-DCs generated a significantly higher percentage of Granzyme B producing CD8^+^ T cells, with a 2.2-fold increase in the MSLN-PDL1-GMCSF-DCs group and had a 2.5-fold increase in the monoclonal antibody group compared to the PBS-DCs group (**Figure 5B**). The similar trend was observed for perforin secretion, which was increased by 3.5-fold in the MSLN-PDL1-GMCSF-DCs group and approximately 5.4-fold upregulation in the monoclonal antibody group (**Figure 5C**). These results suggest that DC-targeted MSLN-PDL1-GMCSF vaccine induces an efficient anti-tumor CTL response, which is even more significant in combination with PD-1 McAb.

### Therapeutic Vaccination With DC Loading MSLN-PDL1-GMCSF Vaccine Significantly Inhibits Tumor Growth

**Figure 6:**
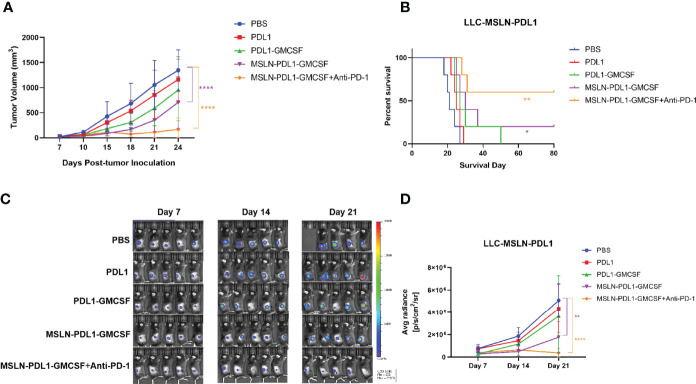
Vaccination combined with PD-1 McAb blockade can synergize with DC-targeted tumor vaccines to control tumor growth. Groups of mice (5 per group) were inoculated subcutaneously with 2×10^5^ exponentially growing and fluorescein-expressing MSLN^+^ and PD-L1^+^ LLC-luc tumor cells. Seven days later, mice were immunized twice (1×10^6^ cells/mouse) with protein-loaded DCs at an interval of 1 week. For combination treatment, PD-1 McAb (200ug/mouse) was injected intraperitoneally in the monoclonal antibody group on day 10 and day 17 after tumor inoculation. Tumor size was measured by vernier calipers every 3-5 days, tumor growth curve is shown in panel **(A)** and survival curve is shown in panel **(B)**. **(C)** Bioluminescence detection of LLC-luc was monitored every seven days. **(D)** Average radiance (photons/second/square centimeter/steradian) of tumor is determined by Living Image 4.5 software. Mean ± SD (n=5). *p < 0.05, **p < 0.01, ****p < 0.0001.

In order to assess whether the fusion protein-loaded DC vaccine can generate therapeutic antitumor effects, 2×10^5^ lung cancer cell lines LLC, which stably expressing MSLN and PD-L1, were injected subcutaneously on the right flank of C57BL/6 mice for better later treatment and monitoring. After tumor establishment, tumor-bearing mice were immunized with fusion protein-loaded DC vaccine *via* footpad injection on day 7 after tumor cell inoculation and booster immunization on day 14. Meanwhile, combination PD-1 monoclonal antibody treatment was administered on days 10 and 17. Tumor growth was monitored by vernier calipers every 3 days, and live imaging was performed every 7 days. As shown in **Figure 6A**, vaccination with MSLN-PDL1-GMCSF-DCs more efficiently delayed tumor growth compared to other groups. Interestingly, immunized mice in the PDL1 protein group and the PDL1-GMCSF vaccine group also showed some efficacy in inhibiting tumor growth. In addition, the survival of tumor-bearing mice was significantly improved by vaccination with MSLN-PDL1-GMCSF-DCs (**Figure 6B**). After injection of luciferase, *in vivo* bioluminescence showed that MSLN-PDL1-GMCSF-DCs combined with anti-PD-1 group greatly inhibited tumor growth *in vivo* (**Figures 6C, D**). Thus, these data suggest that this MSLN-PDL1-GMCSF vaccine could be a more potent therapeutic vaccine compared with conventional non-PD-L1 DC-targeting protein vaccines. Strikingly, Vaccination of PD-1 McAb can synergize with MSLN-PDL1-GMCSF-DCs vaccine to produce significantly enhanced antitumor effect, and 60% of treated mice survived for at least 80 days. Therefore, the combination of protein-loaded DC vaccine and PD-1 McAb blockade could be an effective therapeutic strategy against solid tumors.

## Discussion

In recent years, tumor vaccines designed based on DCs have been extensively studied. DC vaccines loaded with tumor antigens are promising approaches for cancer immunotherapy due to their ability to present specific tumor antigen and activate specific T cells, stimulate B cells and form immune memory (5). Studies have shown that DC vaccines enhance antitumor immune responses and effectively control tumor growth in various mouse tumor models with good safety and tolerability (10, 34, 35). However, despite extensive efforts, tumor regression in DC vaccine-treated cancer patients is rare and limited progress has been made in cancer treatment (6). Therefore, new strategies and approaches are urgently needed to improve the efficacy of tumor vaccines and thus improve the poor efficacy in the clinic. A fusion protein targeting MSLN was found to promote tumor-specific T cell responses by increasing tumor antigen presentation and cross-presentation *via* DC *in vitro* and enhanced tumor cell immunogenicity *in vivo* (36). In this study, we designed a novel fusion peptide immunogen including human MSLN, PD-L1 immune checkpoint molecule, GM-CSF sequence and T helper epitope sequence assembly as therapeutic tumor vaccine (MSLN-PDL1-GMCSF). Our results showed that the novel PD-L1-containing MSLN targeting vaccine was able to activate a MSLN and PD-L1-specific T cell immune responses in immunized mice, inhibit the growth of MSLN^+^ and PD-L1^+^ tumor cells. Meanwhile, cytokine GM-CSF component in the vaccine design as an adjuvant might elicit function in enhancing DC recruitment, activation and cross-presentation.

The immunosuppressive tumor microenvironment leading to inefficient antigen presentation is further complicated by the problem. The immune checkpoint molecule PD-1 is expressed on T cells and its binding with PD-L1 on tumor cells can down-regulate T cell proliferation, reduce T cell cytokine secretion or enhance T cell apoptosis (37–39). To date, there are five antibodies against PD-1 or PD-L1 that have been approved by the FDA and the European Medicines Agency for use in cancer immunotherapy (40). Clinical trials have shown that immune checkpoint regulation therapies have good potential, although in many cases the effect is limited, especially in solid tumors with low response rates, probably because the majority of cancer patients are resistant to PD-1/PD-L1 blockade (41). The induced anti-PD-L1 antibodies can kill PD-L1-expressing tumor cells through multiple mechanisms, including inhibition of PD-L1 interaction with PD-1 on CTLs, antibody-dependent cellular cytotoxicity (ADCC), and complement-dependent cytotoxicity (CDC) (13, 37, 42). Previous evidence supports that DCs can directly induce CTL and antibody responses, and DCs stimulating an antibody response is believed as a consequence of CD4^+^ Th function (13, 43). In this study, we found that this novel PD-L1-containing MSLN vaccine can induce low-dose, durable anti-PD-L1 antibodies and elicit effective tumor-specific CTL responses *in vivo*. As shown in the **Figure 6**, compared with DC vaccine loaded with PD-L1 alone, the MSLN-PDL1-GMCSF vaccine inhibited the growth of lung cancer cells more effectively, and obtained a more pronounced survival benefit, which probably because blocking the PD-1 and PD-L1 restores the anti-tumor effect of T cells in the tumor microenvironment, thus reversing the “cold” state of the tumor.

Several clinical trials are underway to evaluate the efficacy of DC-based vaccines in combination with radiotherapy, chemotherapy, immune checkpoint inhibitors, and adoptive cell therapy for the treatment of various cancers (17, 34, 41). Teng, C. et al. demonstrated that blocking the PD-1/PD-L1 immune checkpoint during DC vaccination showed better therapeutic efficacy than DC vaccination alone in an established mice model of *in-situ* liver tumor (15). However, in contrast to preclinical data, clinical data on the combination of immune checkpoint inhibitors and DCs vaccination are limited. Recent clinical data showed that 39 patients with metastatic melanoma who received ipilimumab plus DCs vaccine had an overall survival rate of only 38% (44). In this study, we further evaluated the therapeutic efficacy of this MSLN-PDL1-GMCSF vaccine in combination with PD-1 immune checkpoint inhibitors in an established mice model of lung cancer. The results showed that by inducing a stronger anti-tumor cytotoxic T-cell response, the combination of MSLN-PDL1-GMCSF vaccine and PD-1 antibody significantly prolonged overall survival, reduced tumor volume and increased tumor cell apoptosis compared to treatment alone. Thus, the combination therapy of this novel vaccine with PD-1 blockade may have great potential as a new treatment strategy for the treatment of other cancers, such as mesothelioma, pancreatic cancer, ovarian cancer and triple negative breast cancer with high expression of MSLN.

Nevertheless, we are aware of some limitations in this study. Firstly, more solid tumors with high expression of MSLN and PDL1 are needed to determine the overall anti-tumor effect of this novel vaccine in the future. Secondly, our next step is to optimize the MSLN epitope sequence to improve immunogenicity. Also, whether this PD-L1-containing MSLN vaccine can efficiently target *in vivo* needs to be determined. In addition, there are some potential clinical applications and challenges for this PD-L1-containing MSLN vaccine. Since the sequences of this novel fusion peptide immunogen (MSLN-PDL1-GMCSF) are originated from human, it is important to test this novel human MSLN-PDL1-GMCSF vaccine in cancer patients to determine whether anti-PD-L1 antibody and CTL responses can be induced and whether the PD-L1-containing MSLN vaccine is safe. Till now immunosuppression microenvironment remain a major challenge in developing effective immunotherapies, the combined therapies (CAR-T and chemotherapy, etc.) that modulate the tumor microenvironment were also needed to enhance the immune response and improve the efficacy of antitumor.

In summary, provided here for the first time is a novel PD-L1-containing MSLN therapeutic vaccine with the ability to induce persistent anti-PD-L1 antibody and CTL responses. It provides a new effective immunotherapeutic strategy for various solid tumors expressing high MSLN and PD-L1 level by combining this MSLN-PDL1-GMCSF vaccine and PD-1 blockade. This work, together with our recent reported PDL1-Vax, lays out a new strategy to overcome the problem of immune tolerance, and promotes the development of drugs targeting MSLN as the relevant antigen, which have theoretical and application values in the development of tumor immunotherapy in human. The combination of therapeutic vaccination with PD-1 blockade therapy may prove to be critical for improving the management and clinical outcomes of patients who do not respond or whose disease eventually progresses.

## Data Availability Statement

The original contributions presented in the study are included in the article/**Supplementary Material**. Further inquiries can be directed to the corresponding authors.

## Ethics Statement

The animal study was reviewed and approved by Guangzhou Medical University.

## Author Contributions

All authors listed have made a substantial, direct, and intellectual contribution to the work and approved it for publication.

## Funding

This work was supported by the research funding the Grant No. 31770183 from the National Natural Science Foundation of China, by the High-level university foundation of the Grant No. JCXKJS2021B04 from Guangzhou Medical University, and supported by the funds under Grant Number 15001019002204 from the Sixth Affiliated Hospital of Guangzhou Medical University, Qingyuan People’s Hospital. This work was also supported by Grant Number A2019144 from Medical Science and Technology Research Foundation of Guangdong Province.

## Conflict of Interest

The authors declare that the research was conducted in the absence of any commercial or financial relationships that could be construed as a potential conflict of interest.

## Publisher’s Note

All claims expressed in this article are solely those of the authors and do not necessarily represent those of their affiliated organizations, or those of the publisher, the editors and the reviewers. Any product that may be evaluated in this article, or claim that may be made by its manufacturer, is not guaranteed or endorsed by the publisher.

## Abbreviations

ADCs, Antibody-drug conjugates; APCs, Antigen-presenting cells; BSA, Bovine Serum Albumin; CTL, Cytotoxic T lymphocyte; DC, Dendritic cell; ELISA, enzyme linked immunosorbent assay; FACS, Fluorescence-activated cell sorting; FBS, Fatal Bovine Serum; GM-CSF, Granulocyte macrophage colony stimulating factor; IFN, Interferon; IL, Interleukin; IPTG, Isopropyl-β-D-1-thiogalactoside; LLC, Lewis lung carcinoma; LPS, Lipopolysaccharide; McAb, Monoclonal antibody; MHC, Major histocompatibility complex; MOI, Multiplicity of infection; MSLN, Mesothelin; PD-1, Programmed death 1; PD-L1, Programmed death ligand 1; PMA, phorbol ester; qPCR, Real-time Quantitative polymerase chain reaction; SDS-PAGE, sodium dodecyl sulfate-polyacrylamide gel electrophoresis; TAAs, Tumor-associated antigens; Th1/Th2, T helper type 1/T helper type 2.
