# Supplementary material for: A Novel PD-L1-Containing MSLN Targeting Vaccine for Lung Cancer Immunotherapy
Source: Front Immunol. 2022 Jun 20;13:925217. doi: 10.3389/fimmu.2022.925217 (PMC9251065; doi:10.3389/fimmu.2022.925217)
Supplement: Supplementary file 1 [file DataSheet_1.docx]

**Supplementary Figure 1**


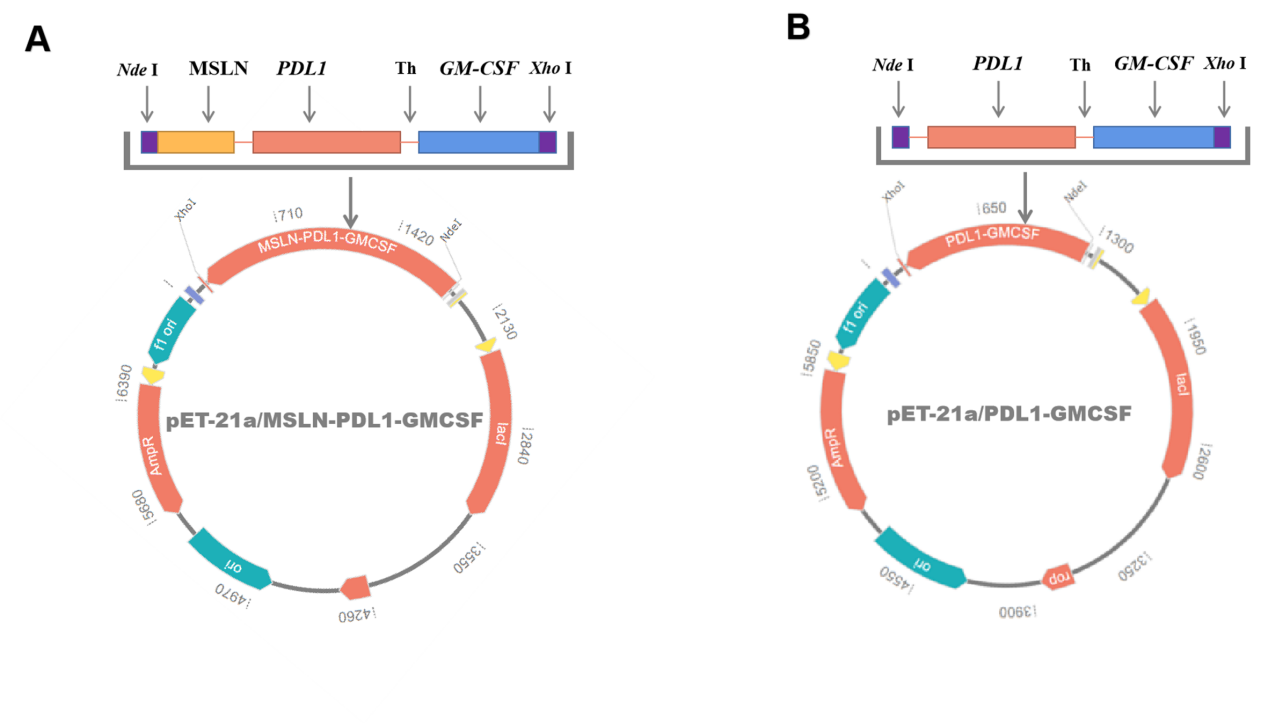


**C DNA sequence of MSLN-PDL1-GMCSF fusion gene**

5’-

CATATGGAGGTTGAAAAAACCGCGTGCCCGAGCGGCAAGAAAGCGCGTGAGATCGATGAAAGCCTGATTTTCTACAAGAAATGGGAGCTGGAAGCGTGCGTGGATGCGGCGCTGCTGGCGACCCAGATGGATCGTGTTAACGCGATCCCGTTTACCTATGAACAACTGGACGTGCTGAAGCACAAACTGGATGAACTGTACCCGCAGGGCTATCCGGAGAGCGTGATCCAACACCTGGGTTACCTGTTCCTGAAGATGAGCCCGGAAGACATTCGTAAATGGAACGTTACCAGCCTGGAGACCCTGAAGGCGCTGCTGGAAGTGAACAAAGGTCATGAGATGAGCCCGCAGGTGGCGACCCTGATCGATCGTTTCGTTAAGGGTCGTGGCCAACTGGACAAAGATACCCTGGACACCCTGACCGCGTTTTACCCGGGTTATCTGTGCAGCCTGAGCCCGGAGGAACTGAGCAGCGTGCCGCCGAGCAGCATTTGGGCGGTTCGTCCGCAGGACCTGGATACCTGCGACCCGCGTCAACTGGATGTTCTGTACCCGAAGGCGCGTCTGGCGTTTCAAAACATGAACGGCAGCGAATATTTCGTGAAGTTTACCGTGACCGTTCCGAAAGATCTGTACGTGGTTGAGTATGGTAGCAACATGACCATCGAATGCAAGTTCCCGGTGGAGAAACAACTGGACCTGGCGGCGCTGATTGTTTACTGGGAGATGGAAGATAAGAACATCATTCAGTTTGTGCACGGCGAGGAAGACCTGAAAGTTCAGCACAGCAGCTATCGTCAACGTGCGCGTCTGCTGAAAGACCAGCTGAGCCTGGGTAACGCGGCGCTGCAGATCACCGACGTGAAACTGCAAGATGCGGGTGTTTACCGTTGCATGATCAGCTACGGTGGCGCGGATTATAAGCGTATTACCGTGAAAGTTAACGCGCCGTATAACAAAATCAACCAGCGTATTCTGGTGGTTGACCCGGTTACCAGCGAGCACGAACTGACCTGCCAAGCGGAAGGCTATCCGAAGGCGGAAGTGATCTGGACCAGCAGCGATCACCAGGTTCTGAGCGGTAAGACCACCACCACCAACAGCAAGCGTGAGGAAAAACTGTTCAACGTTACCAGCACCCTGCGTATCAACACCACCACCAACGAGATTTTCTACTGCACCTTTCGTCGTCTGGACCCGGAGGAAAACCACGCGAAGTTTGTTGCGGCGTGGACCCTGAAAGCGGCGGCGGGTTCGAACGGTAGCGGTAGCGGCATGTGGCTGCAGAGCCTGCTGCTGCTGGGTACCGTTGCGTGCAGCATTAGCGCGCCGGCGCGTAGCCCGAGCCCGAGCACCCAGCCGTGGGAACACGTGAACGCGATTCAAGAGGCGCGTCGTCTGCTGAACCTGAGCCGTGATACCGCGGCGGAGATGAACGAAACCGTTGAGGTGATCAGCGAAATGTTCGACCTGCAGGAGCCGACCTGCCTGCAAACCCGTCTGGAACTGTACAAGCAAGGCCTGCGTGGTAGCCTGACCAAGCTGAAAGGTCCGCTGACCATGATGGCGAGCCACTATAAACAGCATTGCCCGCCGACCCCGGAGACCAGCTGCGCGACCCAAATCATTACCTTCGAGAGCTTTAAGGAGAACCTGAAAGACTTCCTGCTGGTGATTCCGTTTGATTGCTGGGAACCGGTTCAGGAGCTCGAG-3’

**D DNA sequence of PDL1-GMCSF fusion gene**

5’-

CATATGTTTACTGTAACAGTTCCCAAAGATCTATATGTAGTTGAGTATGGCAGCAACATGACCATTGAATGTAAATTCCCGGTGGAGAAGCAACTGGACCTGGCAGCCCTGATTGTTTATTGGGAGATGGAAGACAAAAATATCATTCAGTTTGTTCACGGCGAAGAGGACCTGAAGGTGCAGCACAGCAGCTACCGTCAGCGTGCTCGCCTCCTTAAGGACCAGCTGAGCCTGGGAAACGCCGCTTTACAGATTACCGATGTTAAGCTGCAAGATGCGGGCGTGTACAGATGCATGATTAGTTACGGCGGTGCAGATTATAAGCGCATCACCGTGAAAGTTAACGCGCCATACAACAAGATCAATCAGCGTATCCTGGTTGTCGATCCGGTTACGTCTGAACACGAGTTGACGTGCCAGGCAGAGGGTTATCCGAAAGCGGAAGTTATCTGGACCTCCTCCGATCACCAGGTTCTGTCTGGCAAAACCACCACTACGAATAGCAAACGCGAAGAGAAACTTTTCAACGTGACCTCTACCCTGCGCATCAACACCACGACCAACGAGATCTTCTACTGCACCTTCCGCCGTTTGGACCCTGAGGAGAACCATGCGAAATTCGTGGCGGCCTGGACCCTCAAGGCGGCGGCGGGTTCCAATGGTTCCGGCAGCGGCATGTGGCTGCAAAGCCTGTTGCTGCTGGGTACAGTTGCCTGCAGCATTAGCGCTCCAGCACGTTCTCCGAGTCCGAGCACCCAGCCGTGGGAACACGTGAACGCGATTCAAGAAGCTCGTCGTTTGTTGAATCTGAGCCGTGACACCGCTGCGGAGATGAACGAAACGGTGGAGGTCATCTCCGAAATGTTTGATCTGCAAGAGCCGACCTGCCTGCAGACCCGTTTGGAATTGTACAAGCAAGGTCTGCGTGGTTCGCTCACGAAACTGAAGGGTCCGCTGACCATGATGGCAAGCCATTATAAACAACATTGTCCGCCGACACCGGAAACTTCATGCGCGACCCAAATTATCACCTTTGAAAGCTTTAAGGAAAATCTGAAAGATTTTCTGTTGGTGATCCCGTTCGACTGTTGGGAACCGGTTCAGGAGCTCGAG-3’

**Supplementary Figure 1. Construction of MSLN-PDL1-GMCSF and PDL1-GMCSF.** (A) MSLN-PDL1-GMCSF plasmid construction map. (B) PDL1-GMCSF plasmid construction map. (C) DNA sequence of MSLN-PDL1-GMCSF fusion gene. (D) DNA sequence of PDL1-GMCSF fusion gene.

**Supplementary Figure 2**

**A**


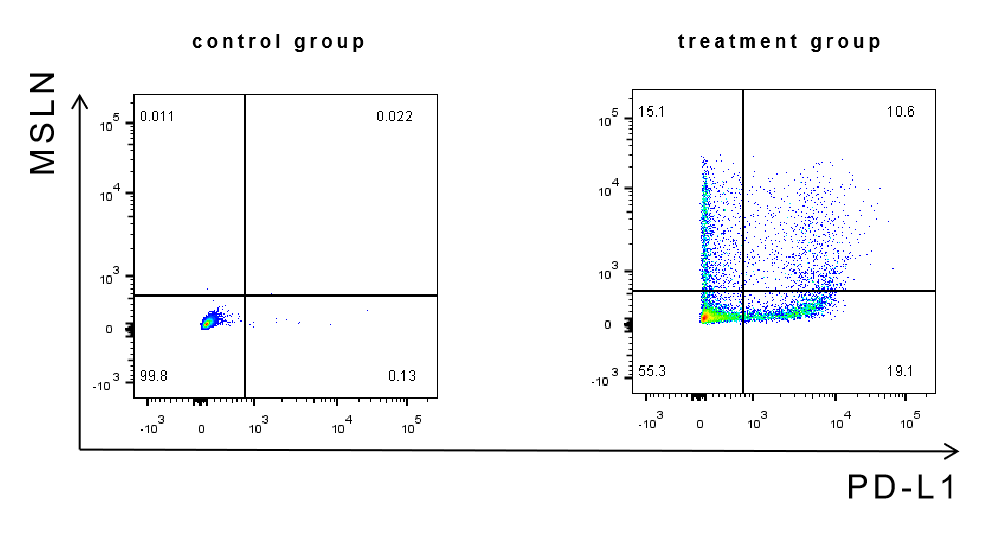


**B**


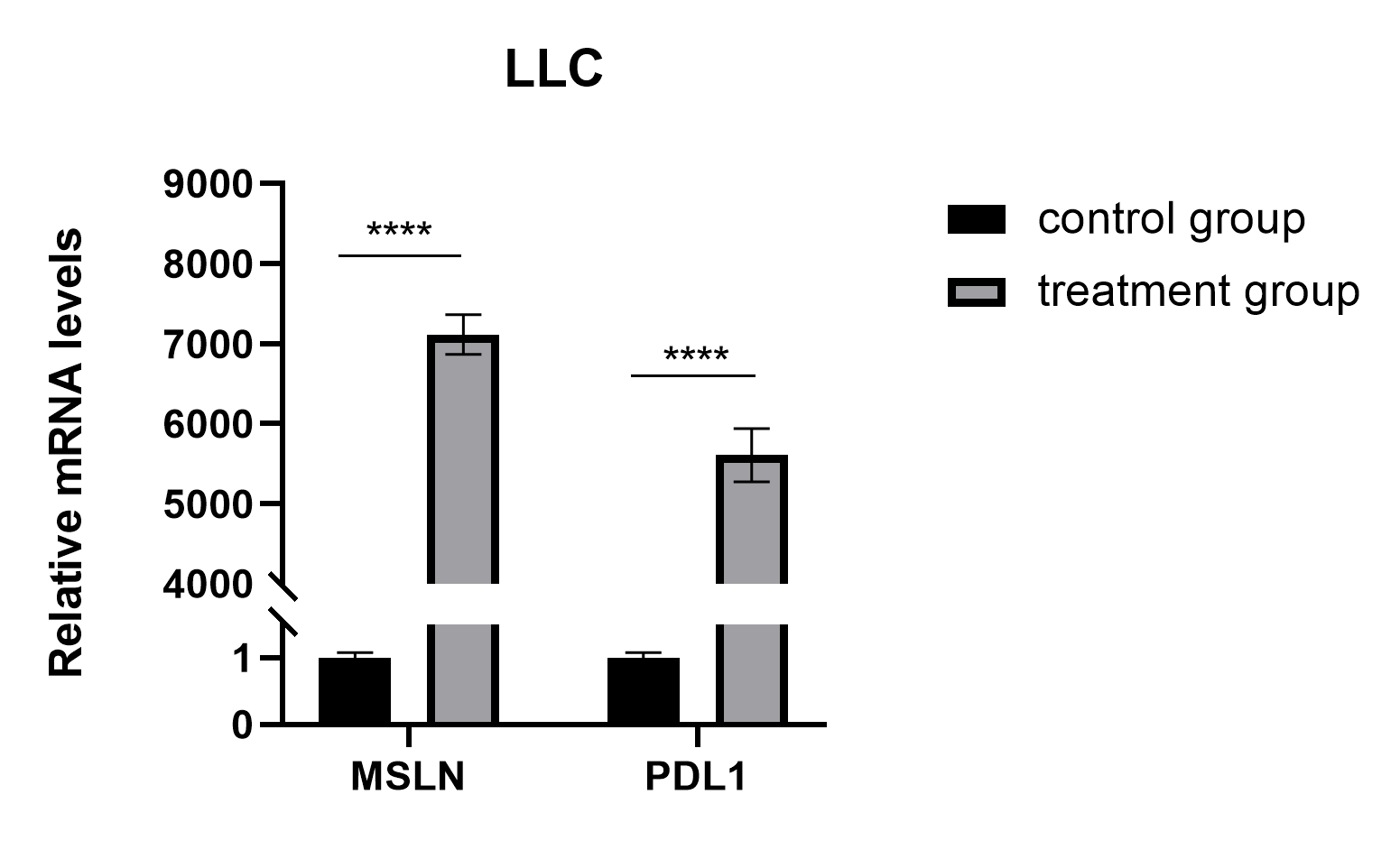


**Supplementary Figure 2. Detection results of LLC tumor cell lines stably expressing MSLN and PD-L1.** The MSLN and PD-L1 were cloned into the lentiviral vector pCDH-CMV-MCS-EF1a-LUC-T2A-Puro and then transfected into the target cells LLC. Puromycin was used to screen for stable cell lines expressing MSLN and PD-L1. (**A**) LLC cells in the treatment group stably expressed MSLN and PD-L1 compared to the control group determined by cell surface staining and flow cytometry. **(B**) The expression of target genes of the RNA level was detected by qPCR. ****p<0.0001
